# The Effects of Implementing a “Waterfall” Emergency Physician Attending Schedule

**DOI:** 10.5811/westjem.2021.2.50249

**Published:** 2021-07-20

**Authors:** Lindsey Spiegelman, Maxwell Jen, Danielle Matonis, Ryan Gibney, Soheil Saadat, Sangeeta Sakaria, Alisa Wray, Shannon Toohey

**Affiliations:** * University of California Irvine Medical Center, Department of Emergency Medicine, Orange, California

## Abstract

**Introduction:**

Increases in emergency department (ED) crowding and boarding are a nationwide issue resulting in worsening patient care and throughput. To compensate, ED administrators often look to modifying staffing models to improve efficiencies.

**Methods:**

This study evaluates the impact of implementing the waterfall model of physician staffing on door-to-doctor time (DDOC), door-to-disposition time (DDIS), left without being seen (LWBS) rate, elopement rate, and the number of patient sign-outs. We examined 9,082 pre-intervention ED visits and 8,983 post-intervention ED visits.

**Results:**

The change in DDOC, LWBS rate, and elopement rate demonstrated statistically significant improvement from a mean of 65.1 to 35 minutes (P <0.001), 1.12% to 0.92% (P = 0.004), and 3.96% to 1.95% (P <0.001), respectively. The change in DDIS from 312 to 324.7 minutes was not statistically significant (P = 0.310). The number of patient sign-outs increased after the implementation of a waterfall schedule (P <0.001).

**Conclusion:**

Implementing a waterfall schedule improved DDOC time while decreasing the percentage of patients who LWBS and eloped. The DDIS and number of patient sign-outs appears to have increased post implementation, although this may have been confounded by the increase in patient volumes and ED boarding from the pre- to post-intervention period.

## INTRODUCTION

Emergency department (ED) crowding and boarding have increased in recent years, a concern that has gained the attention of the media, physicians, and patients. It has been deemed a serious health issue^1^ because patients depend on the ED for access to care for urgent or emergent issues especially when other healthcare options are unavailable.^2^ Additionally, boarding and crowding have significantly strained physicians, healthcare staff, and ED beds, leading to worsened patient outcomes attributed to increased wait times, elopement, and leaving against medical advice.^3^ An issue closely tied to ED crowding is the increase in patient hand-off events that occur when patients remain in the ED for a prolonged period of time (ie, longer than any individual physician’s shift duration). This is problematic as transfers of care have been shown to be the highest risk event for errors in patient care.^4^,^5^

Despite these factors, EDs are continuously attempting to improve performance as measured by metrics such as door-to-doctor time (DDOC) and doctor-to-disposition time (DDIS), as they are correlated with patient satisfaction and clinical quality outcomes.^6^ It has been found that as DDOC increases, there is an increase in the number of patients who leave without being seen (LWBS).^6^ Furthermore, LWBS patients are more likely to present later with a more severe stage of illness and with a higher chance for admission, further straining hospital systems’ limited resources.^6^

One approach to mitigate the negative effects of boarding, potentially decrease patient handoffs, and improve efficiency is to implement a so-called “waterfall” schedule. A waterfall schedule is one where there are overlapping physician shifts. In addition, the model often has physicians changing locations partway through their shifts to be primarily responsible for evaluating different types of patients at different times.^5^ A previous study found that implementing a waterfall schedule demonstrated a “25% reduction in proportion of encounters with patients handoffs…and a survey of physicians and charge nurses demonstrated improved perception of patient safety, ED flow and job satisfaction.”^5^ To determine whether a waterfall schedule could improve flow we instituted a waterfall attending schedule at our ED in February 2018. In this study we evaluate whether implementation of this scheduling model improved ED operational metrics such as DDOC, DDIS, the number of patients who LWBS or eloped, and the number of physician handoffs.

## MATERIALS AND METHODS

This study was performed at a medium-sized, urban, academic ED with a three-year emergency medicine (EM) residency program composed of 24 residents, 20 full-time educational faculty attendings, and 5–8 per diem attending physicians. The hospital is a Level I trauma center, a designated stroke center, and a STEMI, burn, and psych receiving center. The ED has 36 beds, six trauma/resuscitations bays, and up to 20 hallway/chair spaces, and has approximately 54,000 patient visits per year.

### Schedule Format

The pre-waterfall attending physician schedule consisted of two morning shifts (6 am–4 pm and 9 am–7 pm), two afternoon shifts (3 pm–12 am and 6 pm–3 am) and one single-coverage overnight shift (11 pm–7 am) for a total of 46 hours of attending coverage. Additionally, there were two physician-in-triage (PIT) shifts (10 am–6 pm and 5 pm–1 am) whose role was to screen patients as they arrived to the ED and expedite the ordering of labs and imaging while patients waited for examination and treatment spaces to become available. Including the PIT shifts, there was a total of 62 hours of attending coverage.

In February 2018 an attending “waterfall” schedule was implemented based on the model described by Yoshida et al.^5^ Shifts were scheduled from 6 am–3 pm, 9 am–6 pm, 11 am–9 pm, 2 pm–12 am, 5 pm–1 am, 8 pm–4 am, and 11 pm–7 am. The waterfall schedule has 62 hours of attending coverage. There were no entire PIT shifts. All emergency physicians (EP) began their shifts by seeing all new patients arriving to the ED until the next attending arrived to relieve them. With the new schedule, the attending was stationed in triage for the first 2–3 hours of his or her shift with the goal of evaluating every walk-in patient. On initial evaluation the EP could start a note. If time permitted, they could perform an entire history and physical (H&P). If it was particularly busy, they could perform an abbreviated H&P with the plan to re-evaluate the patient again later. Patients were evaluated on an exam table that could be flattened to enable a complete exam.

**Population Health Research Capsule**

What do we already know about this issue?

*Previous research has evaluated the effects of advanced practice providers, fast tracks, and adjustments to physician scheduling to improve emergency department throughput*.

What was the research question?

*We evaluated the effect of a waterfall schedule on door-to-doctor and door-to-disposition times, left without being seen and elopement rates, and number of patient sign-outs*.

What was the major finding of the study?

*The waterfall schedule improved door-to-doctor time, left without being seen rates, and elopement rates*.

How does this improve population health?

*Physician scheduling can expedite patient care and decrease elopement and left without being seen rates*.

**Figure 1:**
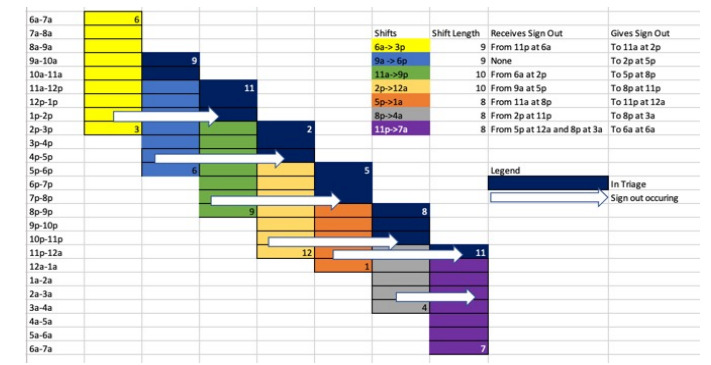
Waterfall schedule for emergency physician attendings.

Once relieved, the EP would sign out to another physician. Afterward they would transition to become the “back doctor” and would see ambulance runs while dispositioning the patients who had been initially triaged. Specifically, the 6 am–3 pm EP signs out to the 11 am–9 pm EP, the 9 am–6 pm EP signs out to the 2 pm–12 am doctor, the 11a-9p doctor signs out to the 5 pm–1 am EP, and the 2 pm–12 am doctor signs out to the 8 pm–4 am EP. Both the the 5 pm–1 am and 8 pm–4 am EPs sign out to the 11 pm overnight physician at 12 am and 3 am, respectively (Figure 1).

Advanced practice providers (APP) continued to manage fast-track patients with low Emergency Severity Index levels between 10 am–7 pm, as they had prior to the implementation of the attending waterfall schedule. The calculations for DDOC time and DDIS time included fast-track patients for pre and post implementation.

### Data Collection

Aggregated de-identified data was extracted from the electronic health record (Epic Systems Corporation, Verona, WI) for the periods before implementation (December 1, 2017–January 31, 2018) and after implementation (December 1, 2018–January 31, 2019) of the waterfall schedule. The institutional review board deemed the study to be exempt from review. We excluded February 1–November 30, 2018 to account for inconsistencies and confounders associated with the transition. We evaluated DDOC times, DDIS times, number of attending sign-outs, number of patients who eloped, and number of patients who LWBS.

### Statistical Analysis

We excluded the highest 1% DDOC times and DDIS times, in order to remove extreme outlier values. The one-sample Kolmogorov-Smirnov test was used to examine the distribution of DDOC and DDIS. None of them followed normal distribution; therefore, we used non-parametric Mann-Whitney U tests to compare DDOC and DDIS before and after the intervention. A *P*-value <0.05 was considered statistically significant. We used SPSS Statistics version 26 for Windows (IBM Corporation, Armonk, NY) for data analysis.

## RESULTS

The study included 9083 charts before and 8983 charts after the intervention. There were 49.9% females in the pre-implementation group and 50.1% in the post-implementation group. The average age was 48.7 in the pre-implementation group and 48.6 in the post-implementation group. The overall department demographics and make-up did not change between pre and post implementation. Refer to Table 1 for demographics.

Table 1 shows the distribution of DDOC and DDIS before and after the intervention, excluding the top 1% extreme values. The change in DDOC was statistically significant from a mean of 65.1 to 35 minutes (*P* <0.001). However, the change in DDIS from 312 to 324.7 minutes seemed to reflect a slight increase although not statistically significant (*P* = 0.310). Excluding the top 1% did not change the statistical significance of DDOC. Excluding the top 1% did make the DDIS lose statistical significance. We excluded the top 1% regardless of its effect on the results because these 1% are outliers that do not represent the bulk of patients (Table 2).

There were 102 LWBS in the pre group (total N: 9083) and 64 LWBS in the post group (total N = 8983) implementation. The prevalence of LWBS was 1.12% in the pre-implementation group and 0.92% in the post-implementation group (*P* = 0.004). A total of 360 patients eloped in the pre and 175 eloped in the post group. The prevalence of elopement was 3.96% in the pre- and 1.95% in the post-implementation group (*P* < 0.001).

**Figure 2:**
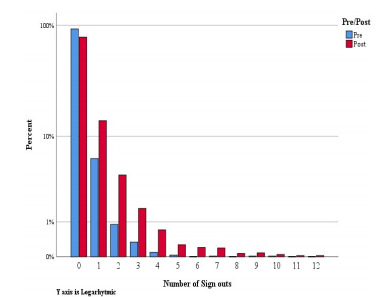
Pre- and post-implementation sign-outs.

Figure 2 shows the number of sign-outs pre and post implementation. The number of sign-outs skewed toward a higher number in the post group as compared to pre (*P* <0.001) The average number of sign- outs was 0.1 in the pre-implementation group and 0.4 in the post group.

**Figure 3:**
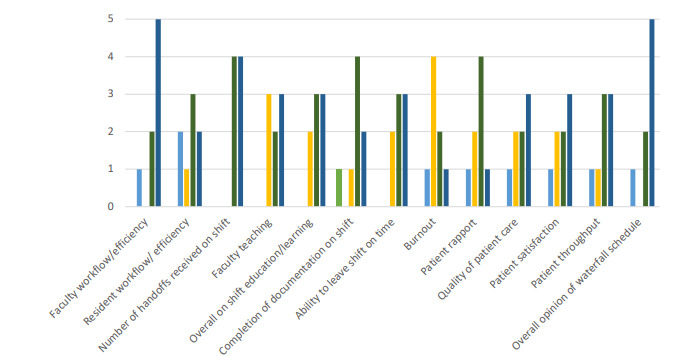
Attending survey results and response rate; N = x/y.

We conducted a post-implementation survey of the attending physicians and received eight responses. Of those eight responses, seven were faculty before and after implementation. Of the eight attendings, three were formerly residents. The survey inquired about the attending’s opinion of the waterfall schedule’s effect on faculty workflow, resident workflow, number of handoffs, faculty teaching, on-shift education, on-shift documentation, ability to leave shift on time, burnout, patient rapport, quality of patient care, patient satisfaction, patient throughput, and overall opinion. These results are summarized in Figure 3.

## DISCUSSION

Given the recent emphasis on increased ED efficiency and throughput, studies have begun to evaluate how different physician staffing and patient distribution models are improving these metrics. It appears that using PIT doctors, fast track and APPs is an improvement, yet there may be ways to improve throughput even further. In one study an ED changed its attending staffing model from non-overlapping shifts to overlapping shift times and noted that door-to-full-exam time decreased from 84 minutes to 52 minutes without increasing staff hours.^7^ Another study compared using a PIT doctor to moving that physician to the main ED without changing physician staffing hours and found improvement in DDOC time, DDIS time, and decreased LWBS.^8^ An important study that inspired ours was performed by Yoshida et al. They implemented a waterfall schedule where a new attending arrives every 3–5 hours. When the new attending arrives, he or she sees new, high- acuity patients until the next attending arrives and he or she transition to a secondary role where they disposition their patients and see lower acuity patients. Yoshida and colleagues found a 25% reduction in patient handoffs but no improvement in median length of stay.^5^ The waterfall schedule we implemented was slightly different than Yoshida’s. Ours has EPs seeing a high volume of lower acuity patients during their triage time, and then transitioning to the higher acuity ambulance runs at the end of the shift. The patients who are “triaged” during the beginning of the shift remain the attendings’ patients throughout the entirety of their shift, as opposed to other triage models in which another physician would primarily manage and follow up on results.

At our institution we already had a PIT doctor and a fast track staffed by APPs. Yet due to the significant patient volumes and ED boarding, our ED staff suffered from significant delays in patient throughput. By the afternoon all ED beds were full, and patients were getting the majority of their treatment in the waiting room. The PIT doctor was therefore responsible for the patients who had been triaged until they were placed in a main ED bed, which was often many hours later. Attendings function at different speeds, and there was significant variation in patient volumes seen depending on which attending was assigned to triage. Benefits of the PIT model were extremely variable. Some PITs would screen 40+ patients and discharge 10+ while others would screen under 20 and discharge none, resulting in significant, variable downstream impacts including leaving the ED attendings with 20–30+ pending patients. Some attendings were proactive about discharging patients who had complete workups while they were still in the waiting room while others did not. Furthermore, the addition of another physician to the traditional academic center model of the resident-attending physician team led to patient confusion over who his or her doctor was. Furthermore, orders placed in triage were often not consistent with what the attending EP wanted and would result in the overutilization of resources, a known issue with PIT systems.^9^,^10^

Our goals in implementing the waterfall schedule were to standardize the process and minimize variability in patient volumes. The idea of having a PIT doctor is sound, yet new information shows that having one provider be primarily responsible for a patient could be more efficient.^9^ This waterfall schedule could potentially represent the best of both worlds – an attending designated to triage for quick evaluation and maintain the patient’s continuity of care team throughout the ED stay.

Our study found a statistically significant improvement in DDOC while a non-statistically significant increase in DDIS. In addition, we found a statistically significant improvement in rates of both patients who LWBS and elopements. The improvement in DDOC, LWBS rates, and decreased number of elopements is consistent with previous studies.^2^,^13^ It is possible that the decreased LWBS in the post group could have increased the DDIS time, as previously these patients were not waiting for care. Furthermore, seven of the eight attendings we surveyed reported that the waterfall schedule positively or strongly positively improved faculty workflow and efficiency. Furthermore, six of the eight felt that this new schedule positively or strongly positively improved patient throughput. We suspect the lack of significant change in DDIS was likely related to the overall hospital model. Since our institution is a medium-sized teaching hospital, residents in the ED as well as on consulting services play a significant role in the disposition of patients. Changes to an attending schedule, therefore, might not have as notable an effect on ED metrics as they would at non-teaching hospitals. Significant increases in delays are often related to our consultant responses.

In discussion with physicians and other ED staff we found that morale and patient care improved after implementation of this new model. Three of eight faculty responded that the waterfall schedule either positively or strongly positively impacted their burnout. In addition, five of eight faculty believed the new schedule either positively or strongly positively improved patient satisfaction as well as the overall quality of the patient’s care. The attendings considered prior ED PIT shifts to be extremely stressful and overwhelming. In the survey one attending wrote, “the triage shifts were horrible!” Additionally, the triage shifts did not significantly decrease patients per hour for the other EPs who ultimately still evaluated the patients once beds were open. One attending commented in the survey that a main benefit of the schedule change was that, “two attendings didn’t need to talk to the patient…that patients are seen and followed by the same attending.” When asked what was the worst part of the new waterfall schedule their answers were that “the shifts are very front loaded…seeing the majority of your patients in the first 2–3 hours can be tough and you have to move quickly.” Despite comments about the shift being frontloaded, the overall sense is that the scheduling changes improve burnout and physician satisfaction as the triage shifts are stressful and challenging. The current model is much improved because the designated triage time is limited to three hours as opposed to an entire shift.

Additionally, attendings take full responsibility for all patients seen during their shifts, allowing the patients to be spread out among seven attendings per day instead of five, with two attendings only providing initial evaluation and orders. Furthermore, by placing the triage time at the beginning of the shift, physicians are able to disposition their patients more, and hand-offs subjectively seem to be better. Our results interestingly skewed toward having more sign-outs post implementation. Yet, all eight survey results stated that the number of hand-offs received on shift after implementation of the waterfall schedule was either positive or strongly positive with several comments about being able to disposition patients by the end of shift and having fewer hand-offs. There were no responses indicating that sign-outs were worse after implementation.

We believe the disparity in the numerical data and the survey data is related to the increased boarding and increased psychiatric population. These patients who are admitted or waiting for psychiatric placement often remain in the ED for up to 20, or even 60, hours and are signed out by too many EPs. So, while active sign-outs decreased, overall sign-outs of admitted patients increased, thereby affecting the numeric results. In addition, as compared to the Yoshida model, our waterfall schedule requires EP attendings to see emergency medical services runs at the end of their shifts. These patients tend to be more complicated and therefore often have longer lengths of stay. This could also partially explain the increased number of sign-outs.

This study is significant because it is the first to evaluate this kind of attending staffing model at a teaching hospital with EM residents. The previously implemented PIT model did not allow for residents to initially evaluate patients and obtain the “first touch.” In the prior model residents would wait to see patients who would be placed in main ED beds, which often occurred after their labs and imaging had resulted. Studies have evaluated how a PIT doctor affects resident education. One study evaluating the impact of a PIT doctor via a questionnaire found there was a negative impact on development of a differential diagnosis and an emphasis on disposition as compared to an emphasis on initial evaluation.^11^ The waterfall model helps negate this issue. As the PIT will be the physician of record, residents come to triage and perform the initial assessment with the attending.

When the waterfall attending schedule was initiated, the resident schedule remained unchanged. Residents either had a morning shift, a swing shift, or a night shift. Times varied slightly by postgraduate year (PGY) level. At any given time, there was one resident from each PGY level in the ED. The residents were not assigned to a particular attending. They were instructed to evaluate ambulance runs primarily and when time permitted to evaluate triage patients with the triage attending. Residents were in triage initially evaluating patients around 70% of the time. They could then formulate a plan and coordinate with the attending as they would both continue the patients’ care even when they moved to the back. Although the post-implementation physician survey had a low response rate, the feedback we did get was generally positive. Five of eight faculty believed the waterfall schedule positively or strongly positively impacted the ability for faculty to teach. Six of eight felt that the change positively or strongly positively improved overall shift education and resident learning.

Future studies could further evaluate the waterfall schedule. First, it would be important to see the impact on a community ED that is attending run to further evaluate the change in disposition time. In addition, looking at the number of sign-outs while controlling for psychiatric patients or admitted ED boarding patients and focusing on only active patients would be an important next step. In addition, evaluating a waterfall schedule for residents in coordination with a waterfall schedule for attendings and the effect on resident learning and efficiency would be another valuable avenue of research.

## LIMITATIONS

One limitation of this study was that it was performed at a single, medium-sized teaching hospital, which makes the findings less generalizable to larger academic centers or community sites. In addition, our institution transitioned to Epic EHR on November 4, 2017, which could have confounded our findings. Another limitation is the lack of specific data in pre- and post-cohorts on admission rates and the number of psychiatric patients. Further, we excluded the top and bottom 1%, which affected our statistical significance and could be considered a limitation. Yet we believe the 1% were outliers and did not represent the majority of our patient population and would therefore not accurately affect our conclusions if those outliers had been left in the sample.

A final limitation is our poor survey response rate and the concern for response bias. As the majority of our responses were positive, it is possible that only those physicians with a particularly positive experience would have taken the time to complete the survey.

## CONCLUSION

Patient volumes and boarding in the ED continue to increase, and staff are attempting to find solutions to improve throughput. Models including PIT doctors, fast track and utilization of APPs show promise; yet implementing specific attending schedules should be considered as well. Our study evaluated the implementation of a waterfall attending schedule at an urban, academic emergency department and showed significant improved in door to doctor time, and the rates of elopement and patients who left without being seen, while there was no significant change in doctor to disposition time.

## Supplementary Information



## Figures and Tables

**Table 1:** Demographic statistics for pre- and post-implementation

	Study phase			

	Pre		Post	
	
	Count	%	Count	%
Gender				
Female	4,531	49.9%	4,499	50.1%
Male	4,551	50.1%	4,484	49.9%
Age group				
≤ 10	20	0.2%	12	0.1%
11 – 20	351	4.3%	409	5.0%
21 – 30	1,451	17.7%	1,457	17.8%
31 – 40	1,424	17.4%	1,316	16.0%
41 – 50	1,263	15.4%	1,202	14.7%
51 – 60	1,361	16.6%	1,443	17.6%
61 – 70	1,096	13.4%	1,158	14.1%
71 – 80	641	7.8%	675	8.2%
81 – 90	423	5.2%	384	4.7%
91 – 100	172	2.1%	143	1.7%
101+	5	0.1%	3	0.0%
Race				
White	6,314	69.5%	6,209	69.1%
Asian	1, 11	12.2%	1,121	12.5%
Black or African American	357	3.9%	360	4.0%
Native Hawaiian or Other Pacific Islander	50	0.6%	55	0.6%
Other/unknown	1,250	13.8%	1,238	13.8%
Ethnicity				
Non-Hispanic [8]	4,986	54.9%	4,995	55.6%
Hispanic [9]	4,006	44.1%	3,904	43.5%
Other/unknown	90	1.0%	84	0.9%

**Table 2:** Distribution of door-to-doctor times (DDOC) and door-to-disposition (DDIS) times in minutes (both excluding top 1%) before and after intervention.

	Before	After
DDOC excluding top 1%		
N	8,482	8,682
Minimum	0	0
Maximum	345	307
Mean	65.1	35
Median	35	24
SD	72.85	33.7
DDIS excluding top 1%		
N	8,588	8,723
Minimum	0	1
Maximum	3,891	4,353
Mean	312	324.7
Median	209	211
SD	352.12	445.65

*SD*, standard deviation.
