# Supplementary material for: The Effects of Implementing a “Waterfall” Emergency Physician Attending Schedule
Source: West J Emerg Med. 2021 Jul 20;22(4):882–9. doi: 10.5811/westjem.2021.2.50249 (PMC8328172; doi:10.5811/westjem.2021.2.50249)
Supplement: Supplementary file 1 [file wjem-22-882-s001.docx]

**Supplemental Table.** Social determinants of health of patients surveyed.

| **Social Determinants of Health with**  **Z-codes** | N | % | **Social Determinants of Health with**  **Z-codes** | N | % |
| --- | --- | --- | --- | --- | --- |
| **Problems related to housing and economic circumstances- Z59** |  |  | **Personal history of psychological trauma - Z91.41** |  |  |
| **Stability of living condition** |  |  | **Ever faced physical, emotional, or verbal abuse?** |  |  |
| Have a steady place to live | 202 | 75.1% | No | 163 | 60.6% |
| Have a place to live but worried about losing it | 27 | 10.0% | Yes | 104 | 38.7% |
| Don't have a steady place to live | 40 | 14.9% | Missing | 2 | 0.7% |
| **Poor living conditions** |  |  | **Problems related to employment - Z56** |  |  |
| No | 68 | 25.3% | **Problems related to employment** |  |  |
| Yes | 201 | 74.7% | Employed -working ≥ 40 hours per week | 62 | 23.0% |
| **Food insecurity or shortage** |  |  | Employed - working 1-39 hours per week | 74 | 27.5% |
| No | 130 | 48.3% | Not employed, looking for work | 46 | 17.1% |
| Yes | 139 | 51.7% | Not employed, not looking for work | 34 | 12.6% |
| **Reliable transportation** |  |  | Retired | 16 | 5.9% |
| No | 184 | 68.4% | Disabled, not able to work | 37 | 13.8% |
| Yes | 84 | 31.2% | **Problems related to social environment - Z60** |  |  |
| Missing | 1 | 0.4% | **Help with day-to-day activities** |  |  |
| **Utilities threatened to be shut off?** |  |  | Don't need help | 171 | 63.6% |
| No | 235 | 87.4% | Get all help that they need | 45 | 16.7% |
| Yes | 34 | 12.6% | Could use some help | 35 | 13.0% |
| **Financial Insecurity** |  |  | Need a lot more help | 17 | 6.3% |
| No | 81 | 30.1% | Missing | 1 | 0.4% |
| Yes | 188 | 69.9% | **Feeling lonely or isolated** |  |  |
| **Substance Abuse** |  |  | Never | 140 | 52.0% |
| **Alcohol** |  |  | Rarely | 36 | 13.4% |
| Never | 179 | 66.5% | Sometimes | 45 | 16.7% |
| Once or twice | 33 | 12.3% | Often | 23 | 8.6% |
| Monthly | 17 | 6.3% | Always | 24 | 8.9% |
| Weekly | 29 | 10.8% | Missing | 1 | 0.4% |
| Almost Daily | 9 | 3.3% | **Mental Health** |  |  |
| Missing | 2 | 0.7% | **Little interest or pleasure in doing things** |  |  |
| **Cigarette** |  |  | Not at all | 172 | 63.9% |
| Never | 180 | 66.9% | Several days | 53 | 19.7% |
| Once or twice | 11 | 4.1% | More than half the days | 12 | 4.5% |
| Monthly | 6 | 2.2% | Nearly everyday | 30 | 11.2% |
| Weekly | 16 | 5.9% | Missing | 2 | 0.7% |
| Almost Daily | 54 | 20.1% | **Feeling down, depressed, or hopeless** |  |  |
| Missing | 2 | 0.7% | Not at all | 142 | 52.8% |
| **Prescription drugs**  **for non-medical reasons** |  |  | Several days | 75 | 27.9% |
| Never | 243 | 90.3% | More than half the days | 18 | 6.7% |
| Once or twice | 14 | 5.2% | Nearly everyday | 32 | 11.9% |
| Monthly | 3 | 1.1% | Missing | 2 | 0.7% |
| Weekly | 2 | 0.7% | **Feel tense, restless, nervous, or anxious, sleepless** |  |  |
| Almost Daily | 5 | 1.9% | Not at all | 83 | 30.9% |
| Missing | 2 | 0.7% | Little bit | 62 | 23.0% |
| **Illegal drugs** |  |  | Somewhat | 38 | 14.1% |
| Never | 214 | 79.6% | Quite a bit | 31 | 11.5% |
| Once or twice | 19 | 7.1% | Very much | 53 | 19.7% |
| Monthly | 7 | 2.6% | Missing | 2 | 0.7% |
| Weekly | 15 | 5.6% | **Difficulty concentrating, remembering, or making decisions** |  |  |
| Almost Daily | 12 | 4.5% | No | 174 | 64.7% |
| Missing | 2 | 0.7% | Yes | 93 | 34.6% |
| **Access to healthcare** |  |  | Missing | 2 | 0.7% |
| **Barrier in accessing healthcare** |  |  | **Problems related to education and literacy - Z55** |  |  |
| No | 87 | 32.3% | **Highest Education** |  |  |
| Yes | 182 | 67.7% | Less than high school | 60 | 22.3% |
| **Type of barrier in accessing healthcare** |  |  | High school or equivalent (GED) | 118 | 43.9% |
| No Insurance | 109 | 40.5% | Higher Education | 90 | 33.5% |
| Not able to afford medical bills | 33 | 12.3% | Missing | 1 | 0.4% |
| Do not know when to go to the doctor | 8 | 3.0% |  |  |  |
| Lack of transportation | 8 | 3.0% |  |  |  |
| Other | 14 | 5.2% |  |  |  |
| Missing | 97 | 36.1% |  |  |  |
| **Reason for coming to the ED** |  |  |  |  |  |
| Felt very sick | 103 | 38.3% |  |  |  |
| Sent to ED by the provider | 53 | 19.7% |  |  |  |
| Not aware of other options | 11 | 4.1% |  |  |  |
| Concerned about cost of other medical facilities | 31 | 11.5% |  |  |  |
| Other | 67 | 24.9% |  |  |  |
| Missing | 4 | 1.5% |  |  |  |

*GED*, general education diploma; *ED*, emergency department.
